# An Intelligent Model Predictive Control Framework for Low-Frequency Seismic Vibration Suppression in Active Isolation Systems

**DOI:** 10.3390/s26092770

**Published:** 2026-04-29

**Authors:** Qiuxia Fan, Ruidong Wang, Zefeng Yan, Qianqian Zhang, Chan Xu, Miaoshuo Li

**Affiliations:** School of Automation and Software Engineering, Shanxi University, Taiyuan 030006, China; fanqiuxia0808@sxu.edu.cn (Q.F.); yanzf@sxu.edu.cn (Z.Y.); zhangqianqian@sxu.edu.cn (Q.Z.); xuchan@sxu.edu.cn (C.X.)

**Keywords:** active vibration isolation, model predictive control, data-driven prediction, seismic disturbance suppression, low-frequency vibration control, multilayer perceptron

## Abstract

Low-frequency seismic disturbances significantly limit the performance of precision engineering systems and active vibration isolation platforms. Model predictive control (MPC) is widely applied in such systems due to its ability to handle multi-variable dynamics and constraints. However, its performance strongly depends on model accuracy. To address this issue, this paper proposes a multilayer perceptron-enhanced model predictive control (MLP-MPC) framework for active vibration isolation. In the proposed approach, a multilayer perceptron (MLP) is trained offline to learn the mapping between the current system state and the free-response term in the MPC prediction equation. During online implementation, the trained MLP replaces the model-based free-response calculation while preserving the original quadratic programming structure of conventional MPC. The proposed method is evaluated on a single-degree-of-freedom active vibration isolation system under low-frequency sinusoidal excitation and measured seismic disturbances. The simulation results show that MLP-MPC achieves reduced running RMS tracking error and lower moving-window RMS error compared with conventional MPC and Proportional–Integral–Derivative (PID) control. The results suggest that integrating data-driven free-response estimation into predictive control provides a practical approach to enhancing the performance of low-frequency vibration suppression while maintaining computational feasibility.

## 1. Introduction

Low frequency micro-vibration is one of the main factors limiting the performance of precision manufacturing systems, aerospace equipment and high-sensitivity scientific instruments [1,2,3,4]. In the ground-based gravity wave detection system, seismic interference is one of the main noise sources that restrict the further improvement of detection sensitivity. With the continuous improvement of the detection accuracy requirements, the suppression ability of the traditional passive vibration isolation technology in the low frequency band becomes insufficient. Therefore, active vibration isolation technology has become a hot research topic in academic and engineering circles. In this context, recent studies have explored adaptive and hybrid control strategies to enhance vibration suppression performance under complex operating conditions [5,6].

Active vibration isolation systems counteract external disturbances by generating control forces in real time, offering significant advantages over passive structures in suppressing low- and mid-frequency vibrations. Among various control strategies, model predictive control (MPC) is regarded as an advanced control method suitable for multi-degree-of-freedom active vibration isolation platforms due to its capability to handle multi-input multi-output systems and explicitly incorporate input and output constraints into the optimization process. MPC has been successfully applied to vibration isolation platforms, demonstrating effective suppression performance under complex operating conditions [7].

However, the practical implementation of MPC in high-performance active vibration isolation systems still faces several challenges. First, the prediction accuracy of MPC strongly depends on the accuracy of the mathematical model of the controlled system. In practical vibration isolation platforms, nonlinearity, parameter uncertainty, and unmodeled dynamics are common. Model mismatch may introduce prediction errors that accumulate over the prediction horizon, thereby degrading control performance. Second, MPC requires solving a finite-horizon constrained optimization problem at each sampling instant. When the system dimension increases or a longer prediction horizon is adopted, the computational burden rises significantly, which may introduce additional computational delays in real-time implementation [8,9,10]. In active vibration isolation systems operating under low-frequency seismic excitation, such time-related prediction and computational delays may directly affect transient tracking accuracy and disturbance suppression capability [11]. Data-driven MPC approaches have also been developed to improve robustness against system uncertainties and external disturbances [12]. Similarly, advanced MPC frameworks incorporating fault tolerance and uncertainty handling have further improved vibration suppression performance in flexible structures [13].

In the field of structural vibration control, magnetorheological (MR) dampers have been widely investigated due to their fast response and semi-active control capability. For structural systems equipped with multiple MR dampers, cooperative control and system optimization are critical issues affecting the overall vibration mitigation performance. Yang et al. [14] proposed an ant colony-inspired cooperative control strategy for a multi-MR damper system to mitigate coupled translational–torsional vibrations in spatially irregular structures. By mimicking the adaptive behavior of ant colonies, the control currents of multiple MR dampers can be dynamically allocated to achieve coordinated vibration suppression. Furthermore, Yang et al. [15] investigated the cross-scale integrated optimization of MR damper control systems for torsional vibration mitigation in spatially irregular structures. By simultaneously optimizing the micro-parameters of MR fluids, the structural parameters of MR dampers, and their layout configuration using a genetic-algorithm-based co-simulation framework, the proposed approach significantly improved vibration mitigation performance while reducing system cost.

In practical engineering applications, PID control remains widely used in active vibration isolation systems. However, under identical system conditions, MPC generally demonstrates superior performance in multi-variable coordination and constraint handling. Cheng-Long Yu et al. proposed an optimized MPC algorithm for a magnetoelectric hybrid low-frequency vibration isolation system, significantly improving computational efficiency [16]. Jiyuan Jiang et al. addressed the dependence of MPC on model accuracy by integrating data-driven techniques with MPC for a quasi-zero stiffness vibration isolation structure, thereby enhancing vibration isolation efficiency [17]. Compared with PID control, MPC can naturally consider the coupling effect in multi-degree-of-freedom systems, and PID usually requires an additional decoupling strategy or feedforward compensation to achieve equivalent performance [18].

In recent years, the integration of machine learning and neural networks into predictive control frameworks has provided new possibilities for reducing model uncertainty and improving prediction accuracy. Neural state-space models have been introduced to improve prediction accuracy and system identification in MPC-based vibration control [19]. Recent work has explored neural-network-based MPC frameworks for nonlinear vibration isolation systems, demonstrating improved prediction capability and control performance [20]. Reinforcement learning-based MPC approaches have also been explored to enhance adaptability and control performance in nonlinear systems [21]. For example, Hamid et al. proposed an online identification and control scheme based on wavelet neural networks combined with MPC, enhancing disturbance rejection capability and reducing dependence on precise mathematical models [22]. Wentao Wu et al. combined machine learning and intelligent materials to improve the sensitivity and accuracy of vibration isolation systems [23]. Guoquan Wu et al. constructed a vibration isolation model using a physics-informed recurrent neural network (PIRNN), providing an accurate predictive model for MPC operation [24]. Jianhua Guo et al. enhanced control response speed and accuracy by combining machine learning with fuzzy MPC [25]. Overall, these studies demonstrate that integrating machine learning into MPC frameworks can effectively enhance prediction accuracy and robustness in vibration control systems. Although these hybrid methods improve the nonlinear approximation ability and anti-interference performance, they usually involve complex training process, structural modification of the original MPC formula or additional stability considerations.

To address the above issues, this paper proposes a multilayer perceptron-enhanced model predictive control (MLP-MPC) method for active vibration isolation under low- and mid-frequency seismic disturbances. In the proposed framework, a multilayer perceptron (MLP) is trained offline to learn the mapping between the current system state and the corresponding free-response term in the MPC prediction equation. During online control, the trained MLP is embedded into the prediction stage of MPC to provide rapid estimation of the free-response component. This approach retains the explicit constraint-handling capability and receding-horizon optimization structure of conventional MPC, while introducing a data-driven prediction enhancement mechanism to reduce numerical accumulation errors in the prediction stage and reduce time-related prediction inaccuracies.

The main contributions of this paper are as follows:

1. A control framework integrating an offline-trained multilayer perceptron with model predictive control is developed, enabling data-driven estimation of the free-response term while preserving the original MPC optimization structure.
2. Comparative simulations under sinusoidal and measured seismic excitations are conducted to evaluate the vibration suppression performance of the proposed method.
3. The performance of the single-degree-of-freedom active vibration isolation platform under low-frequency sinusoidal excitation and measured medium- and low-frequency seismic interference is systematically evaluated. The results show that compared with traditional MPC, MLP-MPC achieves higher tracking accuracy, improved damping performance, and enhanced acceleration suppression capability.

The remainder of this paper is organized as follows. Section 2 introduces the theoretical formulation of the proposed MLP-MPC method. Section 3 presents the simulation setup and comparative performance analysis. Section 4 summarizes the full text and discusses the future research direction.

## 2. Basic Theory and Problem Formulation

### 2.1. Active Vibration Isolation System Modeling

The active vibration isolation platform considered in this study can be described by a lumped-parameter dynamic model. The translational motion of the isolated platform relative to the ground is governed by

(1) $$m\ddot{x}\left(t\right)+c\dot{x}\left(t\right)+kx\left(t\right)=u\left(t\right)+d\left(t\right)$$

where m, c and k denote the equivalent mass, damping coefficient, and stiffness of the system, respectively; $u\left(t\right)$ is the control force generated by the actuator; and $d\left(t\right)$ represents the external ground disturbance.

By defining the state vector as $x_{1}\left(t\right)=x\left(t\right)$, $x_{2}\left(t\right)=\dot{x}\left(t\right)$, the continuous time state space model can be written as

(2) $$\;\dot{x}(t)=\left[\begin{array}{cc} 0 & 1\\ -\frac{k}{m} & -\frac{c}{m} \end{array}\right]x\left(t\right)+\left[\begin{array}{c} 0\\ \frac{1}{m} \end{array}\right]u\left(t\right)$$

(3) $$y\left(t\right)=\left[\begin{array}{cc} 1 & 0 \end{array}\right]x\left(t\right),$$

where $y\left(t\right)$ represents the platform displacement output.

The model is discretized using a zero-order hold method with sampling period h, yielding the discrete-time state-space model

(4) $$\begin{array}{l} x_{k+1}=Ax_{k}+Bu_{k}\\ y_{k}=Cx_{k} \end{array}$$

This discrete-time model serves as the baseline model for the MPC and MLP-MPC controllers.

### 2.2. Conventional Model Predictive Control Formulation

In the conventional MPC framework, the predicted output over a prediction horizon N can be decomposed into two components: the free response caused by the current state and the forced response induced by future control inputs.

The stacked prediction vector can be written as

(5) $$Y={\mathrm{Fx}}_{k}+\Theta U$$

where Y is the predicted output sequence, ${\mathrm{Fx}}_{k}$ represents the free-response term, U is the future control sequence, Θ is the dynamic matrix constructed from A,B,C.

The quadratic cost function is formulated as

(6) $$J={\left(Y-R\right)}^{T}Q\left(Y-R\right)+U^{T}RU$$

where R denotes the reference trajectory and Q and R are weighting matrices.

Substituting the prediction Equation (5) into the cost function (6) yields the quadratic programming form in (7).

(7) $$\underset{U}{\min}\frac{1}{2}U^{T}EU+H^{T}U$$

where the Hessian matrix E and gradient vector H are constructed as

(8) $$\begin{array}{l} E=2\left(\Theta^{T}Q\Theta +R\right)\\ H=2\Theta^{T}Q\left({\mathrm{Fx}}_{k}-R\right) \end{array}$$

At each sampling instant, only the first element of the optimal control sequence is applied to the system, following the receding-horizon strategy.

It should be noted that the prediction accuracy of (6) directly depends on the accuracy of the model matrices A, B, and C. In practical active vibration isolation systems, modeling errors and unmodeled dynamics may cause inaccuracies in the free-response term ${\mathrm{Fx}}_{k}$ which accumulate over the prediction horizon and affect transient response performance.

### 2.3. Multilayer Perceptron-Based Free-Response Estimation

To reduce the dependence of MPC on precise modeling, this study introduces a multilayer perceptron (MLP) to estimate the free-response term in a data-driven manner.

An MLP is a feedforward neural network composed of an input layer, hidden layer and output layer. For the l-th layer, the output is given by

(9) $$z^{\left(l\right)}=\sigma \left(W^{\left(l\right)}z^{\left(l-1\right)}{+b}^{\left(l\right)}\right)$$

where $W^{\left(l\right)}$ and $b^{\left(l\right)}$ denote the weight matrix and bias vector, respectively, and $\sigma \left(\cdot \right)$ is the activation function. In this study, the Rectified Linear Unit (ReLU) is adopted:

(10) $$\sigma \left(x\right)=\max\left(0,x\right)$$

During training, the mean squared error (MSE) loss is used:

(11) $$L=\frac{1}{N_{s}}\sum_{i=1}^{N_{s}}{\left\|{\hat{\;f\;}}_{i}-f_{i}\right\|}^{2}$$

where ${\hat{\;f\;}}_{i}$ denotes the predicted free-response vector of sample i, $f_{i}$ denotes the corresponding target free response obtained from the nominal model, and $N_{s}$ is the number of training samples.

To avoid ambiguity, $f_{i}$ here represents the free-response vector and is distinct from the system output $y_{k}$ defined in (4).

The network parameters are optimized using the Adam algorithm to ensure stable convergence.

### 2.4. MLP-Based Free-Response Estimation

In the proposed method, the MLP is trained offline using simulation-generated state-response data. The trained network establishes a mapping

(12) $$f_{k}=N_{\theta }\left(x_{k}\right)$$

where $N_{\theta }$ denotes the trained MLP with parameters θ, and $f_{k}$ is the estimated free-response vector corresponding to the current state.

During online control, the predicted output becomes

(13) $${Y=f}_{k}+\Theta U$$

The quadratic programming matrices are then constructed as

(14) $$\begin{array}{l} E=2\left(\Theta^{T}Q\Theta +R\right)\\ H=2\Theta^{T}Q\left(f_{k}-R\right) \end{array}$$

This formulation preserves the original optimization structure of conventional MPC while replacing the model-derived free-response term with an MLP-based estimation.

Because the MLP is trained offline and only forward propagation is required during online control, the additional computational cost is limited. Compared with recomputing high-order model-based free-response matrices at each sampling instant, neural-network-based estimation can reduce time-related computational overhead and mitigate prediction inaccuracies caused by model mismatch.

**Figure 1:**
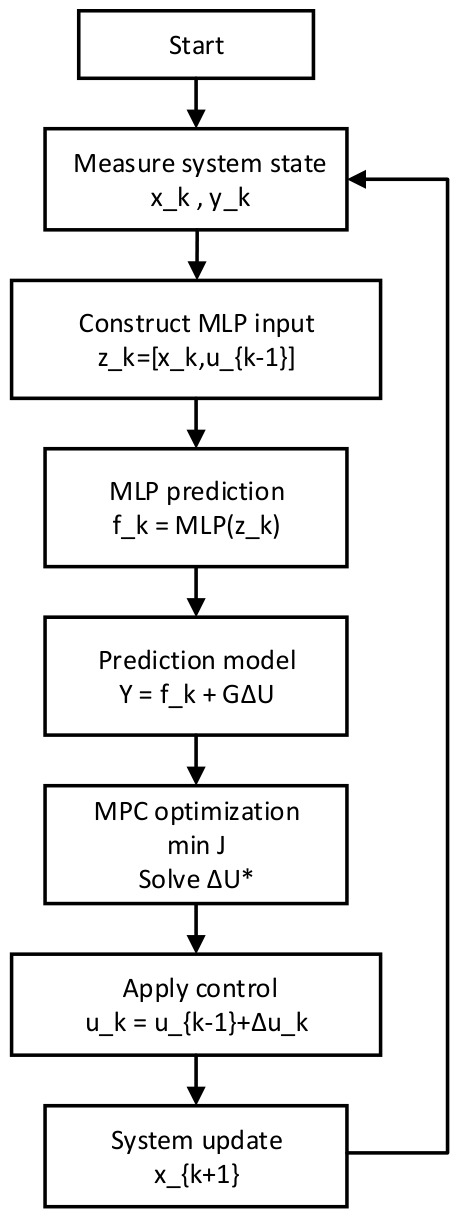
Flowchart of the proposed MLP-MPC control algorithm, where ΔU* represents the optimal control sequence obtained from the MPC optimization problem, and the superscript “*” denotes the optimal solution.

Figure 1 data flow diagram of the proposed MLP-MPC framework. At each sampling instant, the system state $x_{k}$ is measured and fed into the trained MLP to estimate the free-response term $f_{k}$. The estimated free response is then used in the MPC prediction equation, and the resulting quadratic programming problem is solved to obtain the optimal control increment. Only the first element of the optimal control sequence is applied to the system.

### 2.5. Closed-Loop Stability Analysis

The stability of the proposed MLP-MPC framework is analyzed based on the input-to-state stability (ISS) theory.

For a conventional MPC controller, the discrete-time linear system can be written as

(15) $$x_{k+1}=Ax_{k}+Bu_{k}$$

where $x_{k}$ is the system state and $u_{k}$ is the control input.

The optimal control sequence is obtained by minimizing the quadratic cost function defined in (6). The optimal cost value at time k, denoted as $V\left(x_{k}\right)$, can be used as a Lyapunov candidate function.

For the nominal MPC without prediction error, it can be shown that the optimal cost function satisfies

(16) $$V\left(x_{k+1}\right)-V\left(x_{k}\right)\leq -x_{k}^{T}Qx_{k}$$

which indicates that the cost function decreases monotonically along the closed-loop trajectory, implying asymptotic stability of the nominal MPC system.

In the proposed MLP-MPC framework, the free-response term is estimated by a trained neural network rather than being calculated directly from the model. Let the estimation error of the MLP be denoted as

(17) $$e_{k}=f_{k}-{\hat{f}}_{k}$$

where $f_{k}$ is the model-based free response and ${\hat{f}}_{k}$ is the MLP-estimated value.

This estimation error introduces an additional disturbance term in the closed-loop dynamics.

Substituting the perturbed prediction into the state update equation yields

(18) $$x_{k+1}=\left(A+BK\right)x_{k}+w_{k}$$

where K denotes the nominal MPC feedback gain and $w_{k}$ represents the disturbance induced by the prediction error.

According to the input-to-state stability (ISS) theory for discrete-time systems, if the nominal closed-loop system $\left(A+BK\right)$ is stable and the disturbance $w_{k}$ is bounded, then the system state satisfies

(19) $$\left\|x_{k}\right\|\leq \beta \left(\left\|x_{0}\right\|,k\right)+\gamma \left(\underset{i<k}{\sup}\left\|w_{i}\right\|\right)$$

where $\beta \left(\cdot \right)$ is an exponentially decaying function and $\gamma \left(\cdot \right)$ is a bounded gain function.

Since the MLP is trained offline to approximate the free-response mapping and the prediction error remains bounded after convergence, the disturbance term $w_{k}$ is bounded. Therefore, the proposed MLP-MPC closed-loop system is input-to-state stable. The state trajectories converge to a bounded neighborhood around the equilibrium point determined by the approximation error.

When the estimation error approaches zero, the MLP-MPC framework reduces to the nominal MPC controller and asymptotic stability is recovered.

This analysis indicates that the neural-network-based free-response estimation does not destroy the inherent stability structure of the MPC controller, while only introducing a bounded steady-state deviation related to the prediction error.

### 2.6. Computational Cost Analysis

To further demonstrate the engineering feasibility of the proposed method, the computational complexity of the MLP-MPC framework is analyzed.

In conventional MPC, the online computational burden mainly comes from two parts: the construction of the prediction matrices and the solution of the quadratic programming (QP) problem. The complexity of solving the QP problem is approximately $O\left(N_{u}^{3}\right)$ and the complexity of constructing the prediction matrices is approximately $O\left(n^{2}N_{p}\right)$, where n is the state dimension and $N_{p}$ is the prediction horizon. When the prediction horizon is long or the system dimension is high, the matrix power operations become computationally expensive.

In the proposed MLP-MPC framework, the model-based free-response calculation is replaced by a forward pass of the trained MLP. For an MLP with one hidden layer containing h neurons, the complexity of one forward pass is

(20) $$O\left(nh+hN_{p}\right)$$

For a network with two hidden layers, the complexity becomes

(21) $$O\left(nh_{1}+h_{1}h_{2}+h_{2}N_{p}\right)$$

In general, when the state dimension n or the prediction horizon $N_{p}$ is large, the conventional MPC requires repeated matrix power computations with complexity polynomials in both n and $N_{p}$. In contrast, the MLP’s forward propagation involves only simple matrix–vector multiplications with complexity being linear in n and $N_{p}$. As long as the hidden layer sizes h are chosen modestly, the condition $h\ll nN_{p}$ holds and the MLP-based estimation is significantly more efficient than the conventional model-based free-response calculation.

Therefore, compared with conventional MPC, the proposed MLP-MPC reduces the computational burden for long-horizon predictions or high-dimensional systems, offering better scalability for real-time active vibration isolation applications.

## 3. Simulation and Performance Analysis

### 3.1. Simulation Setup

To evaluate the effectiveness of the proposed MLP-MPC method, this paper conducts systematic simulation studies based on the active vibration isolation platform model proposed in [26]. Through comparative analysis with traditional PID control and standard MPC methods, the control performance of the proposed method is comprehensively evaluated under low-frequency and measured low-frequency seismic signals disturbance conditions.

This study focuses on evaluating the effect of data-driven free-response estimation within the classical MPC framework. Therefore, the comparison baseline is limited to PID and conventional MPC. Other advanced control methods, such as adaptive control and sliding mode control, are not considered in this study because the primary objective is to evaluate the computational efficiency and prediction enhancement capability of the proposed MLP-assisted MPC framework under the same predictive control structure.

The simulation sampling period was set to h=0.01 s. The prediction horizon N=10, weighting matrices Q=4000 and R=0.00001, and other MPC parameters were selected to ensure closed-loop stability and a fair comparison among PID, conventional MPC, and MLP-MPC controllers. For consistency, the same system model and control constraints were adopted in both MPC and MLP-MPC implementations.

To comprehensively evaluate the vibration isolation performance of different control methods, two typical simulation scenarios are designed in this paper:

- Low-frequency sinusoidal excitation (frequency of 1 Hz and amplitude of 1 m);
- Measured low-frequency seismic signals imported from an external dataset. (The data were collected by a 731A acceleration sensor (Wilcoxon Sensing Technologies, Germantown, MD, USA). Sampling rate = 2048 Hz, total sampling time = 100 s, unit = V. Samples were collected at the Xiaolianggou Coal Mine.)

The seismic signal was preprocessed and discretized according to the sampling interval of the control system. Performance comparisons were conducted under identical disturbance inputs.

The simulations were implemented in MATLAB R2023b using the Model Predictive Control Toolbox and Neural Network Toolbox.

### 3.2. MLP Training Procedure

The MLP network was trained offline prior to online control implementation. Training data were generated by exciting the system model with various initial states and recording the corresponding free-response vectors used in the MPC prediction equation.

To estimate the free-response term in the MPC prediction equation, a multilayer perceptron (MLP) neural network is constructed as a data-driven approximation model. The MLP is trained offline using the state–response data generated from the vibration isolation system simulation. The network takes the current system state vector as the input and predicts the corresponding free-response component required by the MPC prediction model.

The adopted MLP consists of an input layer, three hidden layers, and an output layer. The hidden layer employs nonlinear activation functions to capture the nonlinear mapping between the system state and the predicted response. The network parameters are optimized through supervised learning using the backpropagation algorithm. After training convergence, the MLP model is embedded into the prediction stage of the MPC controller to provide rapid estimation of the free-response term during online control. The detailed configuration of the adopted MLP network is summarized in Table 1.

The input of the network consisted of the current system state vector $x_{k}$ and the output corresponded to the free-response term over the prediction horizon.

The dataset is divided into training and validation subsets to prevent overfitting. The network is trained using an Adam optimizer with a mean squared error (MSE) loss function and adopts an adaptive learning rate with a maximum of 500 epochs. After convergence, the trained model is saved and embedded into the online control framework.

During online control, only forward propagation of the trained MLP was required to estimate the free-response term. No online parameter updating or retraining was performed.

**Figure 2:**
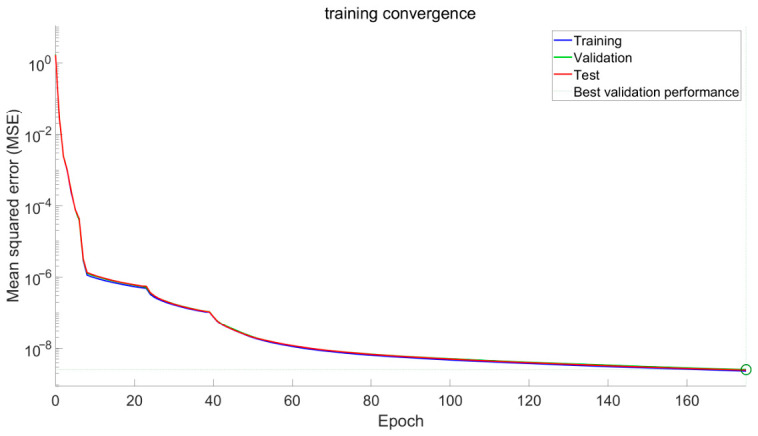
Convergence curves of the MLP network during training, including training, validation, and test errors. The circle indicates the epoch at which the best validation performance is achieved (early stopping point).

The convergence behavior of the MLP network during the training process is illustrated in Figure 2. It can be observed that the loss function gradually decreases and eventually converges, indicating that the network training is stable.

**Figure 3:**
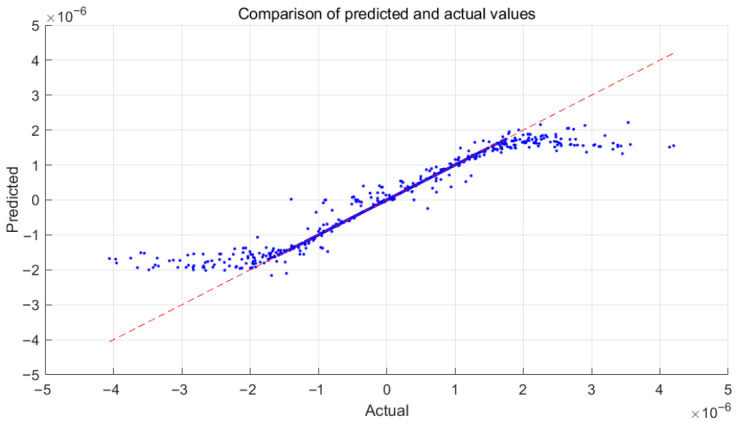
The training performance of the MLP.

**Figure 4:**
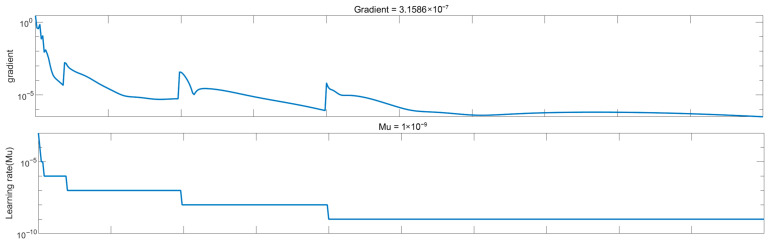
The training progress of the MLP.

Figure 3 shows the predicted versus target values, The data points are closely scattered around the ideal diagonal line, indicating strong correlation between the model outputs and the desired targets. The linear fit further confirms the high predictive accuracy of the trained MLP model. Figure 4 presents the gradient and learning rate curves during training. The gradient decreases to 3.16 × 10^−7^ at epoch 172, while the learning rate reaches 1 × 10^−9^, confirming stable convergence.

It can also be observed that the predicted values exhibit a mild saturation trend at the extreme ranges (approximately beyond ±2 × 10^−6^). This behavior is mainly caused by the limited distribution of training samples near the boundary regions of the dataset. Since the majority of training data are concentrated around the normal operating range of the system, the neural network shows slightly reduced extrapolation capability near the extremes. However, this effect does not influence the control performance because the operational state of the vibration isolation system rarely reaches these boundary regions.

### 3.3. Tracking Performance Under Sinusoidal Excitation

To evaluate basic tracking capability, a low-frequency sinusoidal signal was used as the disturbance input. The responses of PID, conventional MPC, and MLP-MPC were compared.

**Figure 5:**
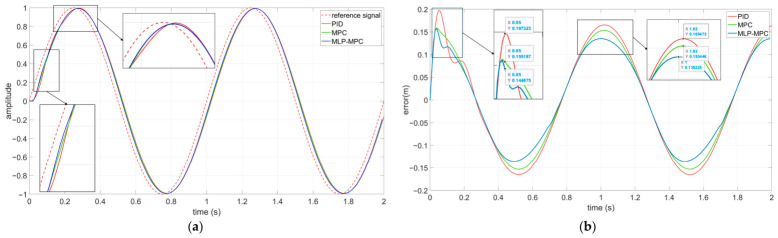
Performance of the three algorithms under low-frequency sinusoidal signals: (**a**) Platform displacement tracking performance of PID, MPC, and MLP-MPC under sinusoidal excitation. (**b**) Platform displacement tracking errors of PID, MPC, and MLP-MPC under sinusoidal excitation.

Figure 5a presents the platform displacement responses under sinusoidal excitation. It can be observed that both MPC-based methods outperform PID control in terms of amplitude suppression and phase alignment. Compared with conventional MPC, the MLP-MPC method exhibits slightly improved transient tracking behavior.

Figure 5b illustrates the tracking error of the three controllers. The MLP-MPC approach achieves reduced peak tracking error and lower RMS error compared with conventional MPC, indicating improved prediction consistency.

Taking the first error peak as an example, MLP-MPC reduces the peak tracking error by approximately 18.3% compared with PID control and by approximately 11.9% com-pared with conventional MPC. The improvement can be attributed to the data-driven estimation of the free-response term, which reduces prediction mismatch during transient periods.

### 3.4. Performance Under Low-Frequency Seismic Disturbances

To further evaluate the disturbance rejection capability under realistic operating conditions, measured low-frequency seismic signals were applied as external excitations. All controllers were implemented under identical simulation settings.

**Figure 6:**
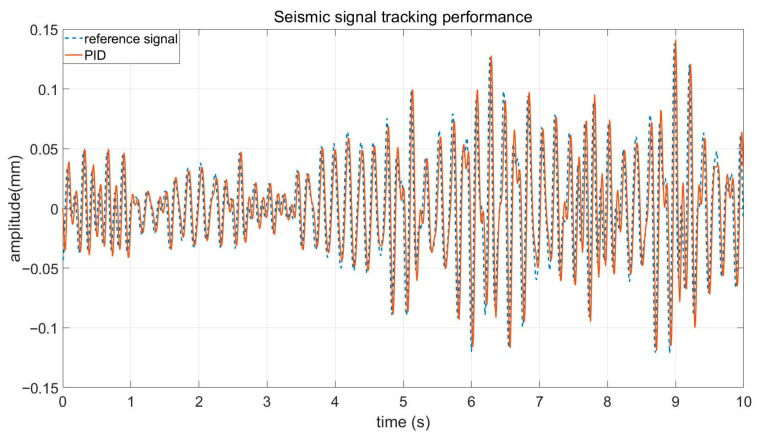

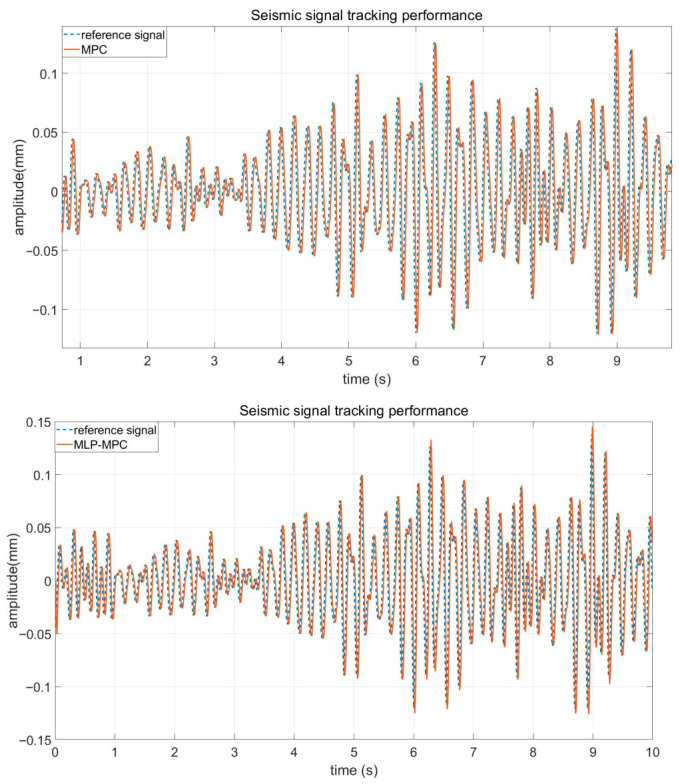
Platform displacement tracking performance of PID, MPC and MLP-MPC under seismic excitation.

Figure 6 shows the platform displacement response of PID, MPC and MLP-MPC under seismic excitation. Compared with PID control, the two predictive control strategies significantly reduce the vibration amplitude. The suppression performance of the MLP-MPC method is slightly improved under sudden disturbance changes.

**Figure 7:**
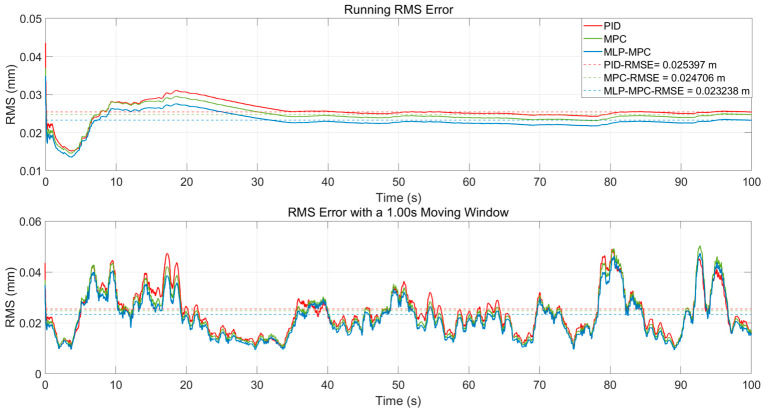
Tracking error (RMSE) profiles of PID, MPC, and MLP-MPC over time.

Figure 7 shows the running RMS errors and the RMS errors calculated using the 1.00 s moving window. These indicators reflect the dynamic accumulation of tracking performance over time and provide a more stable evaluation of disturbance suppression capability.

From the RMS curve, it can be observed that compared with PID control and conventional MPC, the proposed MLP-MPC method maintains a consistently lower error level throughout the entire simulation process. This indicates that the proposed controller achieves a more stable tracking performance under continuous seismic disturbance. The improvement is mainly attributed to the enhanced prediction consistency provided by the MLP-based free-response estimation, which reduces the accumulation of prediction errors over time.

The 1.00 s moving window RMS further highlights the short-term performance differences among the controllers. During rapid disturbance transitions, the RMS error of the conventional MPC temporarily increases due to the mismatch between the model-based prediction and the actual system dynamics. Since the traditional MPC relies entirely on the nominal system model, modeling uncertainties and unmodeled dynamics may lead to prediction deviations during transient disturbance changes. In contrast, the MLP-enhanced method shows a noticeable reduction in peak error accumulation. By learning the mapping between system states and the free-response term, the trained MLP provides a data-driven correction to the model-based prediction, which effectively alleviates short-term prediction bias and improves transient disturbance rejection capability.

**Figure 8:**
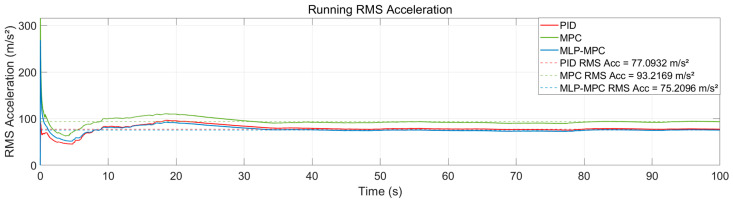

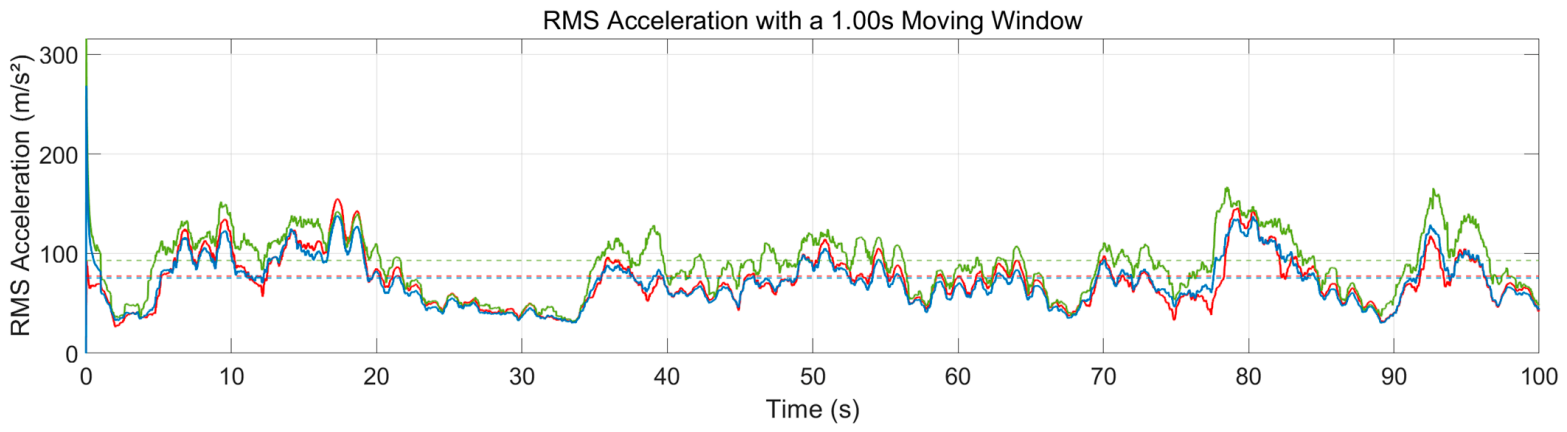
RMS acceleration distribution of PID, MPC and MLP-MPC under seismic excitation.

Figure 8 presents the running RMS acceleration and the RMS acceleration calculated using the 1.00 s moving window. Compared with the conventional MPC controller, the proposed MLP-MPC method further reduces the vibration isolation error and improves the overall acceleration performance of the platform. The reduced RMS acceleration indicates that the controller achieves smoother dynamic responses and better suppression of residual vibrations.

Although the peak acceleration of MLP-MPC is slightly higher than that of PID control, the RMS acceleration level remains significantly lower. This phenomenon can be explained by the different control mechanisms of the controllers. PID control tends to generate smoother but less precise responses due to its limited predictive capability, while predictive controllers actively compensate disturbances through optimization over the prediction horizon.

As a result, MPC-based controllers may occasionally generate larger instantaneous control actions during rapid disturbance changes. However, the improved prediction accuracy introduced by the MLP estimator allows the proposed controller to reduce long-term vibration energy, leading to a lower RMS acceleration level.

Table 2 summarizes the quantitative performance indicators, including maximum tracking error, RMS tracking error, maximum acceleration, RMS acceleration and error 2-norm.

Overall, the results demonstrate that the integration of data-driven free-response estimation into the MPC framework effectively enhances both displacement tracking performance and vibration suppression capability. The proposed MLP-MPC controller achieves improved disturbance rejection while maintaining stable platform dynamics, which is essential for practical active vibration isolation applications.

### 3.5. Spectral Analysis

**Figure 9:**
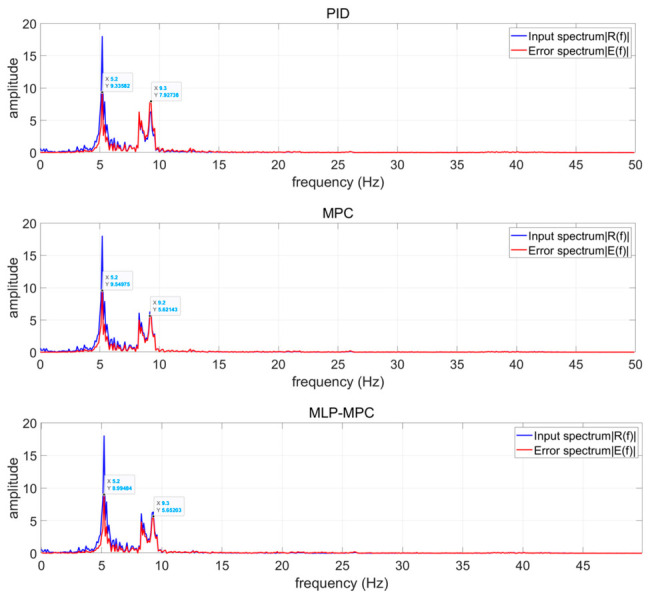
Comparison of error spectra and control input spectra of PID, MPC, and MLP-MPC under low-frequency seismic excitation.

In order to analyze the frequency-domain characteristics of the proposed control strategy, the error spectrum and disturbance spectrum are obtained using the fast Fourier transform (FFT). Figure 9 illustrates the spectral distribution of the disturbance signal and the corresponding tracking errors of different control methods under low-frequency excitation.

It can be observed that the dominant components of the disturbance signal are mainly concentrated in the low-frequency range. Compared with the PID controller, the proposed MLP-MPC method achieves significantly improved attenuation in the low-frequency band. In particular, at approximately 5 Hz, the tracking error amplitude obtained by MLP-MPC is clearly smaller than those produced by both PID and conventional MPC, indicating enhanced disturbance rejection capability in this dominant frequency component. In the frequency range of 8–10 Hz, the proposed MLP-MPC controller still maintains a noticeable advantage over the PID controller, while its suppression performance remains comparable to that of the conventional MPC.

The spectral analysis indicates that the integration of MLP-based free-response estimation mainly improves the prediction accuracy of the control algorithm in the dominant low-frequency region. As a result, the proposed method can more effectively suppress seismic-induced disturbances without sacrificing the stability and frequency response characteristics of the original MPC framework. These results further demonstrate that the proposed MLP-MPC strategy provides improved low-frequency vibration attenuation while maintaining control performance comparable to conventional MPC in other frequency bands.

### 3.6. Robustness Analysis

To further evaluate the robustness of the proposed controller under practical uncertainties, additional simulations were carried out by introducing sensor noise, actuator delay, and parameter perturbations into the system. Specifically, the sensor noise was modeled as zero-mean Gaussian noise with a standard deviation of (1.00 × 10^−7^). The actuator delay was set to one control step to emulate practical control latency. In addition, the system matrices were subjected to parameter perturbations within (±20%) of their nominal values in order to represent possible variations in system dynamics.

**Figure 10:**
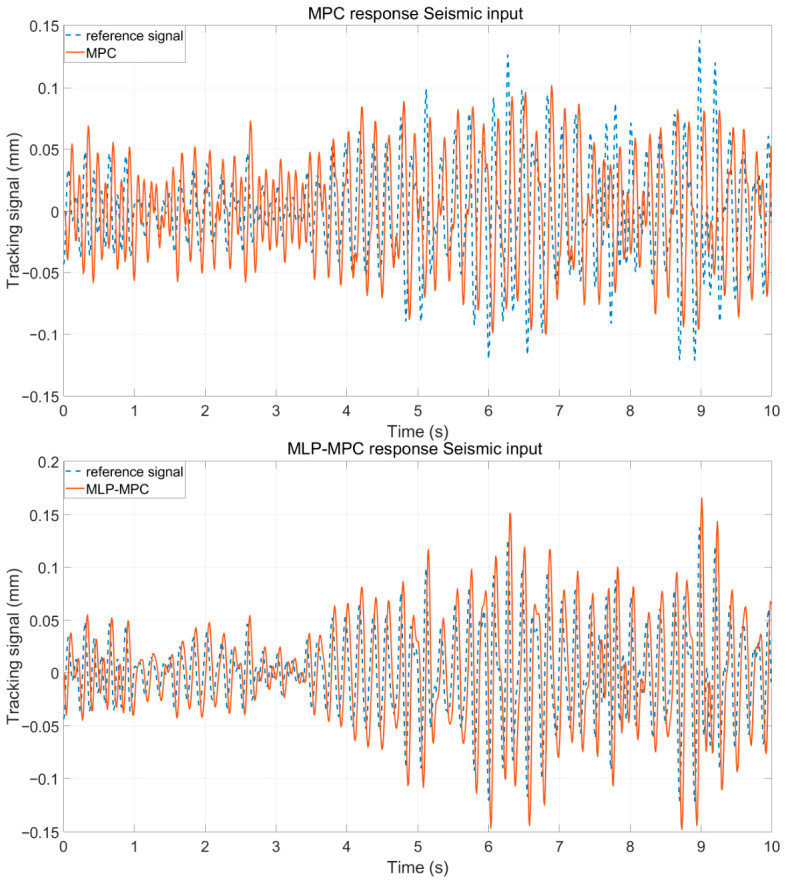
Tracking performance of conventional MPC and the proposed MLP-MPC controller under robustness test conditions with parameter perturbation, sensor noise, and actuator delay.

Figure 10 shows the tracking performance of the proposed MLP-MPC controller under the above robustness conditions. It can be observed that the controller is still capable of effectively suppressing vibration and maintaining stable tracking behavior despite the presence of sensor noise, actuator delay, and parameter uncertainties.

**Figure 11:**
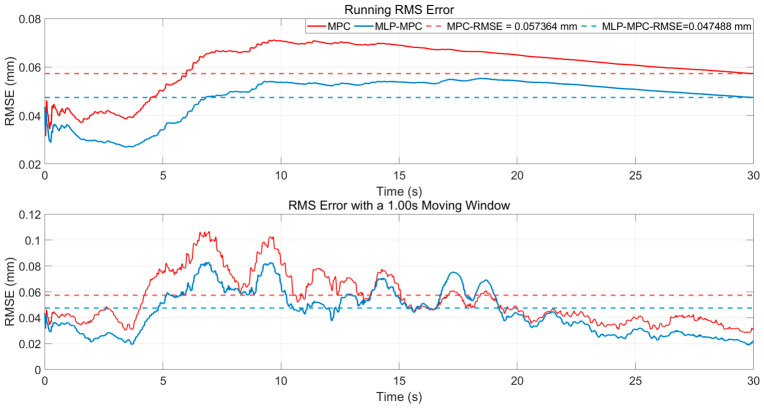
RMS displacement error and RMS error with a 1.00 s moving window for MPC and MLP-MPC under robustness test conditions.

For a quantitative comparison, the RMS displacement errors of the conventional MPC and the proposed MLP-MPC were calculated. The results are illustrated in Figure 11, which presents both the RMS error and the RMS error with a 1.00 s moving window. Under the robustness test conditions, the final RMS displacement error of the conventional MPC reaches 0.057364 mm, whereas the proposed MLP-MPC achieves a significantly lower RMS error of 0.047488 mm.

These results confirm that the proposed MLP-MPC controller remains effective even in the presence of noise, delay, and model uncertainties, highlighting its potential for practical applications in active vibration isolation systems.

### 3.7. Experimental Platform Design for Future Validation

Although the effectiveness of the proposed MLP-MPC method has been verified through extensive numerical simulations, experimental validation is also essential for evaluating its practical applicability. Therefore, an experimental active vibration isolation platform is currently under development to further validate the proposed control framework under realistic operating conditions.

**Figure 12:**
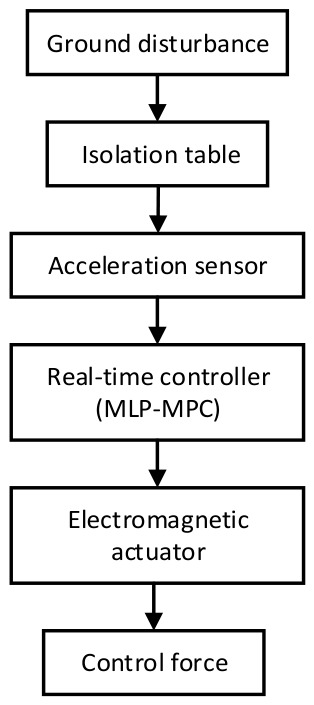
Experimental active vibration isolation platform architecture.

The experimental platform is designed to emulate a typical active vibration isolation system subjected to low-frequency ground disturbances. As illustrated in Figure 12, the system mainly consists of a vibration isolation table, electromagnetic actuators, acceleration sensors, and a real-time control unit. The isolation table is supported by electromagnetic actuators that generate active control forces to counteract external disturbances. High-sensitivity acceleration sensors (PCB 731A, Wilcoxon Sensing Technologies, Germantown, MD, USA) are installed on the isolation platform to measure the vibration response in real time. The measured signals are transmitted to the real-time controller, where the proposed MLP-MPC algorithm is executed.

The control algorithm will be implemented on a real-time control platform based on MATLAB/Simulink Real-Time (version R2023b) and a dedicated real-time processor (such as dSPACE (MicroLabBox) or an equivalent industrial controller). The system sampling frequency is set to 1024 Hz, which is consistent with the sampling rate used in the numerical simulations. The trained MLP model will be embedded into the real-time MPC prediction module to estimate the free-response component during online control.

Future experiments will focus on evaluating the performance of the proposed MLP-MPC controller under practical uncertainties, including sensor noise, actuator delay, and parameter perturbations. In particular, the robustness of the controller will be examined under different noise levels and disturbance conditions, while the influence of actuator dynamics and modeling uncertainties on vibration suppression performance will also be investigated. The experimental results will provide further verification of the feasibility and effectiveness of the proposed MLP-MPC framework for active vibration isolation applications.

## 4. Conclusions

This paper proposed a multilayer perceptron-enhanced model predictive control (MLP-MPC) method for active vibration isolation under low-frequency disturbances. The key idea is to replace the model-based free-response term in the MPC prediction equation with a neural-network-based estimation trained offline, while retaining the standard quadratic programming optimization structure.

The main findings can be summarized as follows:

1. Under sinusoidal excitation, the proposed method achieves better vibration isolation performance, which is mainly reflected at the error peaks;
2. Under measured low-frequency seismic disturbances, the MLP-MPC controller demonstrates consistently lower running RMS error and reduced moving-window RMS error compared with both PID and conventional MPC;
3. The acceleration-related metrics are generally reduced compared with conventional MPC, and the improvements in displacement suppression and tracking consistency indicate that the proposed method achieves both control smoothness and disturbance rejection accuracy.

In general, the framework enhances the consistency of prediction under transient disturbance without modifying the MPC core optimization formula. The offline trained neural network is integrated into predictive control, which provides a practical method to improve the anti-interference performance of low-frequency active vibration isolation systems.

The focus of future work will be to extend the method to a multi-degree-of-freedom platform, study its robustness under the uncertainty of model parameters, and verify the effectiveness of the method through experiments.

## Figures and Tables

**Table 1:** Structure of the adopted MLP network.

Parameter	Value
MLP hidden layers	3
neurons per layer	32-16-8
activation	ReLU
optimizer	Adam

**Table 2:** Performance comparison under low-frequency seismic excitation.

Performance Index	PID	MPC	MLP-MPC	Improvement over PID (%)	Improvement over MPC (%)
Maximum Tracking Error (m)	0.10014	0.09445	0.09003	−10.10	−4.68
RMS Tracking Error (m)	0.025397	0.024706	0.023238	−8.50	−5.94
Maximum Acceleration (m/s^2^)	304.25	395.27	354.08	+16.38	−10.41
RMS Acceleration (m/s^2^)	77.09	93.22	75.21	−2.50	−19.32
Error 2-Norm	0.8845	0.8303	0.7913	−10.54	−4.70

## Data Availability

The original contributions presented in this study are included in the article. Further inquiries can be directed to the corresponding author.
